# Prognosis factors of predicting survival in spontaneously ruptured hepatocellular carcinoma

**DOI:** 10.1007/s12072-022-10403-x

**Published:** 2022-08-25

**Authors:** Peng Wang, Abraham S Moses, Chao Li, Song Chen, Xun Qi, Ke Xu, Hai-bo Shao, Xiang-jun Han

**Affiliations:** 1 Department of Interventional Radiology, The First Affiliated Hospital of China Medical University, Shenyang, 110001 Liaoning China; 2 Department of Pharmaceutical Sciences, College of Pharmacy, Oregon State University, Portland, OR 97201 USA

**Keywords:** Hepatocellular carcinoma, Rupture, Prognosis, MELD, Child–Pugh, Predictive model, Risk factors, Surgical treatment, Transarterial embolization, Survival

## Abstract

**Aim:**

To investigate predictors affecting survival in patients with spontaneously ruptured hepatocellular carcinoma (srHCC).

**Methods:**

One-hundred-and-twenty-seven patients experiencing srHCC between January 2010 and December 2020 were enrolled. The clinical features, treatments, and outcomes were reviewed. Statistics included univariate analysis, Kaplan–Meier analysis, multivariate analysis using Cox proportional hazards model and logistic regression model, and receiver operating characteristic (ROC) curve analysis.

**Results:**

Of the 127 srHCC patients, 24, 42, and 61 patients received conservative treatment, surgical treatment, and transarterial chemoembolization/embolization (TACE/TAE) treatment at HCC rupture, respectively. The largest tumor size [hazard ratio (HR) 1.127; *p* < 0.001], Barcelona-Clinic Liver Cancer (BCLC) stage (HR 2.184, *p* = 0.023), international normalized ratio (INR; HR 3.895; *p* = 0.012), total bilirubin level (TBil; HR 1.014; *p* = 0.014), TACE after rupture (compared with conservative treatment) (HR 0.549; *p* = 0.029), TACE/TAE and surgery at rupture, and albumin level (HR 0.949; *p* = 0.017) were independent predictors affecting overall survival. A survival predictive model for HCC rupture (SPHR) using these predictors was created. ROC analysis showed that the area under the curve (AUC) of the SPHR model for 30 day survival was 0.925, and the AUCs of the model for end-stage liver disease (MELD) score and Child–Pugh score for 30 day survival were 0.767 and 0.757, respectively.

**Conclusion:**

The largest tumor size, advanced BCLC stage, higher INR and TBil, lower albumin, and conservative treatment were negative independent predictors for overall survival. The SPHR model may be more suitable than the MELD score and Child–Pugh score for predicting 30 day survival in srHCC.

**Supplementary Information:**

The online version contains supplementary material available at 10.1007/s12072-022-10403-x.

## Introduction

Hepatocellular carcinoma (HCC) is one of the most common malignant tumors, ranking fifth in incidence and third in tumor-related deaths worldwide [1, 2]. Spontaneous tumor rupture with catastrophic intraperitoneal hemorrhage is a rare and life-threatening complication of HCC, occurring in 2.3–26% of patients with HCC in Asia and less than 3% in the West [3, 4]. A mortality rate of 25–75% is attributed to HCC rupture during the acute phase, with a median survival of 1.2–4 months if untreated [5]. Therefore, identifying prognostic factors and accurately predicting survival will be of great value for patients with HCC rupture. Currently, two scoring systems, the Child–Pugh classification and model for end-stage liver disease (MELD) score, are mainly used for patient counseling, clinical decision-making, and stratifying risk in therapeutic clinical trials [6]. However, as a special condition of HCC rupture, it remains unclear which scoring system has greater predictive value for short-term survival.

Therefore, we conducted the present retrospective study to investigate prognostic factors affecting overall survival in patients with HCC rupture and further investigate the scoring system with greater predictive value in the assessment of 30-day survival after HCC rupture.

## Methods

### Patients

One-hundred-and-twenty-seven patients with spontaneously ruptured HCC were enrolled in our institution between January 2010 and December 2020. The diagnostic criteria of HCC followed Asia–Pacific clinical practice guidelines on the management of hepatocellular carcinoma [7]. Spontaneous rupture of HCCs was diagnosed as abrupt abdominal pain; disruption of the peritumoral liver capsule with enhanced fluid collection in the perihepatic area adjacent to HCC by contrast-enhanced computed tomography (CT), magnetic resonance imaging (MRI) or ultrasound; and hematoma around the liver as revealed by radiological examinations and/or bloody ascites by abdominal paracentesis. Patient data at the time of HCC rupture were recorded, including demographics, hemodynamic status, medical history, tumor characteristics, laboratory data, treatment modality, therapeutic strategies in the follow-up, and survival. This study was conducted in accordance with the Declaration of Helsinki and approved by our institution’s Ethics Committee.

### Treatment

All patients with ruptured HCC immediately received careful conservative treatment, including anti-shock measures and patient condition assessment. Blood biochemical indices and imaging characteristics of HCC were rapidly investigated in the emergency department. Following the evaluation of key variables, including hemodynamic state, tumor status, laboratory data, Child–Pugh score, MELD score, Eastern Cooperative Oncology Group (ECOG) score, and cardiopulmonary function, therapeutic strategies were designed by surgeons, interventional physicians, and patients’ families within 48 h.

### Surgical treatment

The surgical indications included a stable hemodynamic state, satisfactory hepatorenal and cardiopulmonary reservation, and tumor resection or packing. The contraindications included poor liver function (Child C), multifocal HCC, poorly controlled hepatic encephalopathy, severe coagulopathy, main portal vein or hepatic vein invasion, metastasis, and poor heart or lung function. All operations were performed by experienced hepatobiliary surgeons.

### TACE/TAE

Patients contraindicated for surgery were recommended to undergo transarterial chemoembolization/transcatheter arterial embolization (TACE/TAE), and the contraindications included main portal vein thrombosis, arteriovenous fistula, Child–Pugh C cirrhosis, severe coagulopathy, and hepatic encephalopathy. Tumor blood feeding and location were observed through transcatheter hepatic arterial angiography. After a microcatheter was selectively inserted into the feeding tumor artery, embolization was performed with lipiodol, gelatin sponge, or polyvinyl alcohol particles. Common hepatic angiography was then repeated to confirm successful embolization of tumor-feeding arteries.

### Conservative treatment

Patients contraindicated for surgery and TACE/TAE received careful conservative treatments, including intensive care, hemostasis treatment, antishock measures, parenteral nutrition, correction of coagulopathy, and analgesics.

### Follow-up

Follow-up was performed every 1–3 months. Contrast-enhanced CT/MRI, lung CT, liver function, and alpha-fetoprotein levels were evaluated to determine further therapy for these patients. If patients failed to follow up for more than 6 months, the reason was investigated and recorded by doctors via telephone. Overall survival (OS) was defined as the interval from the date of rupture to the date of death or the last follow-up.

### Statistical analysis

Continuous variables were expressed as the means ± SD, and categorical variables were expressed as a number. The survival rate was analyzed using the Kaplan–Meier method, the differences were compared using the log-rank test, and the Bonferroni method was used if more than two factors were included in the analysis. Univariate analysis and multivariate analysis were performed using a Cox proportional hazards model to identify the independent factors of overall survival. Independent factors in multivariate analysis were used to create a new survival predictive model for HCC rupture (hereafter referred to as SPHR) using a logistic regression model. To compare the accuracy of the MELD score, Child–Pugh score, and SPHR model as predictors of 30 day survival, receiver operating characteristic (ROC) curve analysis was conducted to obtain the cutoff value, sensitivity, and specificity. *p* < 0.05 was considered significant. Statistical analyses were performed using SPSS c21.0 software (Chicago, United States) and MedCalc 20.019 software (Los Angeles, United States).

## Results

### Clinical characteristics of spontaneously ruptured HCC patients

One-hundred-and-twenty-seven patients with a diagnosis of spontaneous HCC rupture were enrolled in our study. The mean age of the patients was 55 years. Thirty-eight (29.9%) patients were diagnosed with liver cancer and received TACE treatment before tumor rupture. At the time of rupture, 42 (33.1%), 61 (48.0%) and 24 (18.9%) patients received surgery, TACE/TAE, and conservative treatment, respectively. The surgical management included HCC curative resection (36/42, 85.7%) and perihepatic packing (6/42, 14.3%). Follow-up treatment was started at an average of 13.1 days after HCC rupture, 40 (31.5%) patients received consequent TACE including one plus sorafenib, 87 (68.5%) patients underwent conservative treatment including five cases of sorafenib, no patients received tumor ablation or radiation, and no HCC curative resection or liver transplantation was performed. No patients experienced recurrence of hemorrhage in the follow-up. The mean survival time was 303.0 days, and the median survival time was 165 days. The baseline characteristics and outcomes of the patients are summarized in Table 1.

**Table 1:** Clinical characteristics of patients with ruptured HCC

Variables	*n* (%) or mean ± SD
Age (years)	55.01 ± 11.09
Largest tumor size (cm)	8.39 ± 4.11
Survival time (day)	302.97 ± 380.22
Gender	
Male	106 (83.50)
Female	21 (16.50)
Cirrhosis*	104 (81.90)
Viral hepatitis	
HBV	104 (81.90)
HCV	5 (3.90)
Tumor location	
Left lobe	39 (30.71)
Right lobe	73 (57.48)
Both lobe	15 (11.81)
Rupture location	
Left lobe	45 (35.43)
Right lobe	82 (64.57)
Treatment before rupture	
None	89 (70.08)
TACE	38 (29.92)
Treatment at rupture	
TACE/TAE	61 (48.00)
Surgery	42 (33.10)
Conservative	24 (18.90)
Treatment after rupture	
Conservative	87 (68.50)
TACE	40 (31.50)
Tumor number	
1	57 (44.90)
2–3	35 (27.55)
> 3	35 (27.55)
Tumor size	
< 5 cm	33 (25.98)
≥ 5 cm	94 (74.02)
Up-to-seven**	
≤ 7	34 (26.77)
> 7	93 (73.23)
BCLC stage	
A	10 (7.90)
B	86 (67.70)
C	31 (24.40)
UICC TNM stage	
IIIC	86 (67.72)
IVA	28 (22.05)
IVB	13 (10.23)
Child–Pugh class	
A	67 (52.80)
B	44 (34.60)
C	16 (12.60)
Heart rate	90.99 ± 19.74
AFP, ng/mL	
≤ 400	61 (48.03)
> 400	66 (51.97)
WBC, × 10^9^/L	10.49 ± 5.70
RBC, × 10^12^/L	3.71 ± 0.95
HB, g/L	108.45 ± 25.08
PLT, × 10^9^/L	168.33 ± 86.75
PT, S	15.61 ± 2.94
INR	1.33 ± 0.39
APTT, S	37.81 ± 6.89
PTA, %	74.62 ± 21.14
ALT, U/L	98.86 ± 133.23
ALB, g/L	31.94 ± 7.72
TBil, umol/L	27.53 ± 27.51
K + , mmol/L	4.18 ± 0.60
Cr, umol/L	82.22 ± 41.03
Child–Pugh score	7.11 ± 1.87
MELD score	11.18 ± 4.80
Blood transfusion cases	33(25.98)
Blood transfusion volume (mL)	968.33 ± 702.76
Survival time > 1 month	95 (74.80)
Survival time > 1 year	40 (31.50)

*HBV* Hepatitis B virus, *HCV* Hepatitis C virus, *TACE* Transarterial chemoembolization, *TAE* Transcatheter arterial embolization, *BCLC* Barcelona Clinic Liver Cancer, *UICC* The Union for International Cancer Control, *AFP* alpha-fetoprotein, *WBC* White blood cell count, *RBC* Red blood cell count, *HB* hemoglobin, *PLT* Platelet count, *PT* prothrombin time, *INR* International normalized ratio, *APTT* Activated partial thromboplastin time, *PTA* Prothrombin activity, *ALT* Alanine transaminase, *ALB* Albumin, *TBil* Total bilirubin, *Cr* Creatinine, *MELD* Model for end-stage liver disease

*Cirrhosis was diagnosed by imaging radiologists according to CT, MRI or ultrasound at the diagnosis of HCC

**Up-to-seven: hepatocellular carcinomas with seven as the sum of the largest tumor size (in cm) and the number of tumors

### Univariate analysis for overall survival

Univariate analysis showed that the largest tumor size, tumor number, Up-to-seven criteria, Barcelona-Clinic Liver Cancer (BCLC) C stage, Union for International Cancer Control (UICC) TNM IV stage, prothrombin time (PT) level, international normalized ratio (INR) level, activated partial thromboplastin time (APTT) level, total bilirubin (TBil) level, creatinine (Cr) level, Child–Pugh score and MELD score were significantly associated with poor survival in patients with HCC rupture. In addition, TACE treatment after rupture, prothrombin time activity (PTA) level, and albumin (ALB) level were inversely associated with poor survival. Compared with conservative treatment at rupture, TACE/TAE and surgery were protective factors for patient survival (Table 2).

**Table 2:** Univariate analysis of risk factors related to overall survival for spontaneous rupture of hepatocellular carcinoma

Variables	Patients (*n* = 127)	HR	95% CI	*p*
Age (years)	55.01 ± 11.09	1.006	0.986–1.027	0.541
Gender (male/female)	106/21	1.047	0.796–1.377	0.741
Largest tumor size (cm)	8.39 ± 4.11	1.090	1.039–1.143	< 0.001
Tumor number	2.42 ± 1.55	1.471	1.266–1.710	< 0.001
Tumor size (< 5 cm/ ≥ 5 cm)	33/94	1.572	0.964–2.565	0.070
Up-to-seven (≤ 7/ > 7)	34/93	2.817	1.660–4.783	< 0.001
Tumor rupture location				
Left lobe ( control)	45	–	–	–
Right lobe	82	0.957	0.616–1.485	0.844
BCLC				
A + B (control)	96	–	–	–
C	31	3.111	1.869–5.180	< 0.001
UICC TNM stage				
IIIC (control)	86	–	–	–
IVA + IVB	41	2.213	1.402–3.495	< 0.001
Treatment before rupture				
None	89	–	–	–
TACE	38	0.705	0.445–1.118	0.138
Treatment after rupture				
Conservative (control)	87	–	–	–
TACE	40	0.416	0.253–0.686	0.001
Treatment at rupture				
Conservative (control)	24	–	–	–
TAE/TACE	61	0.345	0.197–0.604	< 0.001
Surgery	42	0.173	0.092–0.324	< 0.001
Virus				
None (control)	18	–	–	–
HBV	104	0.448	0.121–1.660	0.230
HCV	5	0.431	0.132–1.412	0.165
Heart rate	90.99 ± 19.74	1.002	0.990–1.014	0.734
AFP, ng/mL				
≤ 400 (control)	61	–	–	–
> 400	66	1.511	0.976–2.340	0.064
WBC, × 10^9^/L	10.49 ± 5.70	1.006	0.970–1.043	0.758
RBC, × 10^12^/L	3.71 ± 0.95	0.921	0.722–1.174	0.506
HB, g/L	108.45 ± 25.08	0.995	0.986–1.003	0.227
PLT, × 10^9^/L	168.33 ± 86.75	1.001	0.998–1.004	0.568
PT, S	15.61 ± 2.94	1.123	1.042–1.211	0.002
INR	1.33 ± 0.39	3.422	1.736–6.746	< 0.001
APTT, S	37.81 ± 6.89	1.059	1.023–1.096	0.001
PTA, %	74.62 ± 21.14	0.988	0.979–0.998	0.016
ALT, U/L	98.86 ± 133.23	1.001	1.000–1.002	0.102
ALB, g/L	31.94 ± 7.72	0.957	0.929–0.985	0.003
TBil, umol/L	27.53 ± 27.51	1.017	1.010–1.023	< 0.001
K^+^, mmol/L	4.18 ± 0.60	1.061	0.710–1.584	0.774
Cr, umol/L	82.22 ± 41.03	1.006	1.001–1.010	0.014
Child–Pugh score	7.11 ± 1.87	1.273	1.129–1.435	< 0.001
MELD score	11.18 ± 4.80	1.113	1.062–1.166	< 0.001
Blood transfusion volume (mL)	968.33 ± 702.76	0.686	0.416–1.131	0.140

*BCLC* Barcelona clinic liver cancer, *UICC* The Union for International Cancer Control, *TACE* Transarterial chemoembolization, *TAE* Transcatheter arterial embolization, *HBV* Hepatitis B virus, *HCV* Hepatitis C virus, *AFP* alpha-fetoprotein, *WBC* White blood cell count, *RBC* Red blood cell count, *HB* hemoglobin, *PLT* Platelet count, *PT* prothrombin time, *INR* International normalized ratio, *APTT* Activated partial thromboplastin time, *PTA* Prothrombin activity, *ALT* Alanine transaminase, *ALB* Albumin, *TBil* Total bilirubin, *Cr* Creatinine, *MELD* Model for end-stage liver disease

### Multivariate analysis for overall survival

**Fig. 1:**
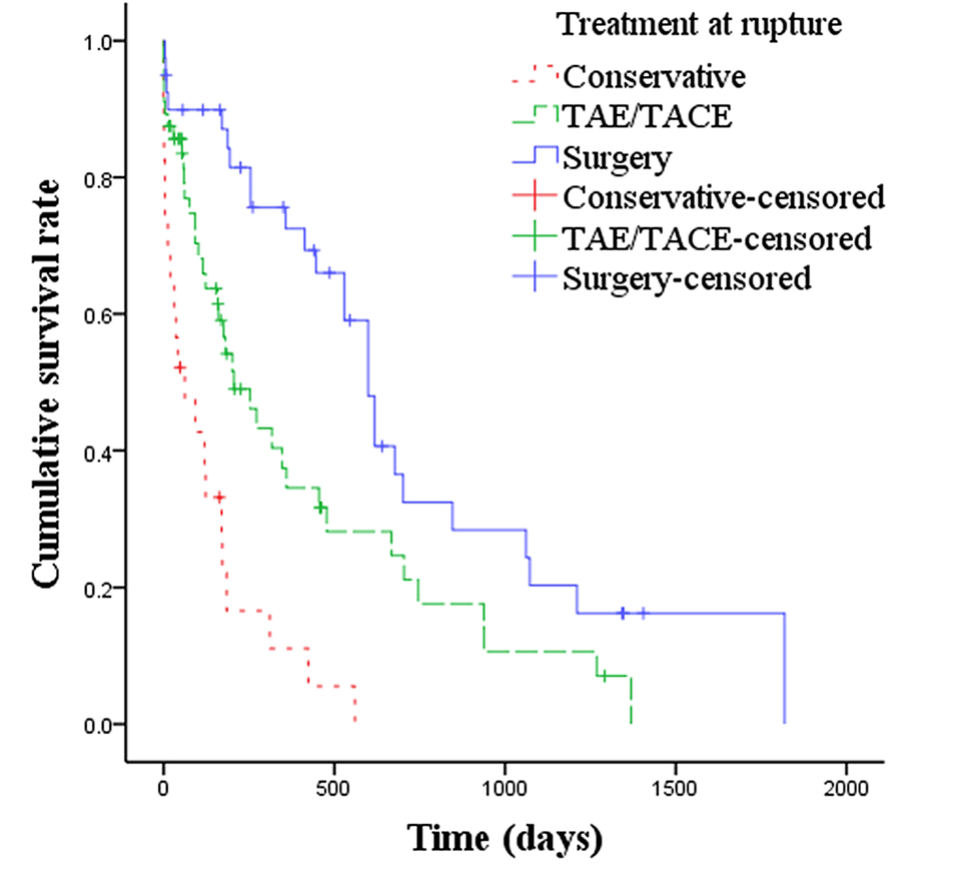
Cumulative survival rate of different therapies at HCC rupture. The cumulative survival rates of patients according to different treatments at HCC rupture were significantly different; The median survival times of conservative, TAE/TACE and surgical treatment were 62 days, 206 days and 599 days, respectively (*p* < 0.001)

**Fig. 2:**
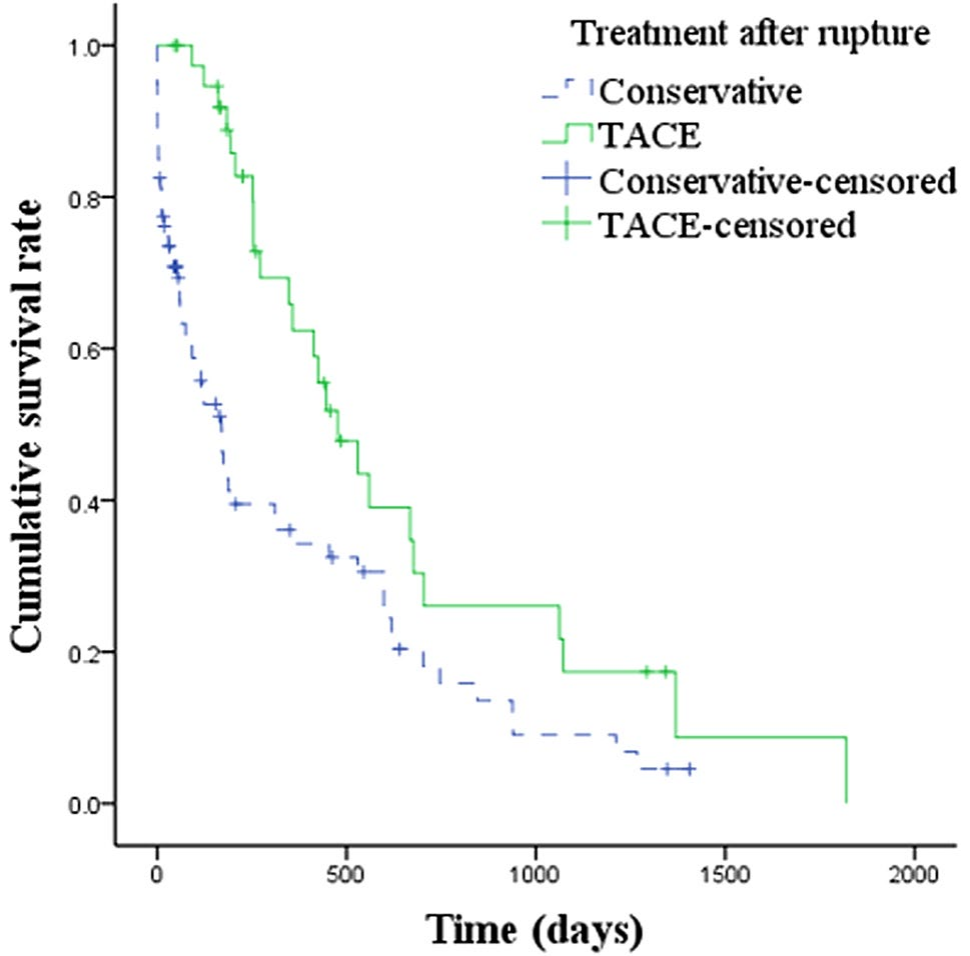
Cumulative survival rate according to different further therapies after hepatocellular carcinoma rupture. Compared with conservative treatment, the cumulative survival rate of patients receiving TACE treatment after HCC rupture was significantly different. The median survival times of conservative treatment and TACE were 170 days and 477 days, respectively (*p* < 0.001)

Stepwise multivariate regression analysis for overall survival was performed following univariate analysis. Up-to-seven criteria and TNM stage were confounding factors for BCLC stage. Child–Pugh score, MELD score and PT were confounding factors for INR and/or TBil. No confounding factors were included in the multivariable analysis (Table 3). The multivariate regression analysis revealed that the largest tumor size [hazard ratio (HR) 1.127; 95% CI 1.056–1.203; *p* < 0.001], BCLC C stage (HR 2.184; 95% CI 1.116–4.276; *p* = 0.023), INR level (HR 3.895; 95% CI 1.344–11.895; *p* = 0.012) and TBil level (HR 1.014; 95% CI 1.003–1.026; *p* = 0.014) were independent risk factors. TACE treatment after rupture (compared with conservative treatment) (HR 0.549; 95% CI 0.321–0.939; *p* = 0.029), TACE/TAE and surgery at rupture, and ALB level (HR 0.949; 95% CI 0.908–0.990; *p* < 0.017) were independent protective factors. The cumulative overall survival rates of ruptured HCC patients with different treatments at rupture differed significantly. The median survival time in patients with conservative treatment, TACE/TAE, and surgery at rupture was 62 days, 206 days, and 599 days, respectively (*p* < 0.001) (Fig. 1). Pairwise comparison using the Bonferroni method showed that differences in conservative vs. TAE/TACE, conservative vs. surgery, and TAE/TACE vs. surgery were also significant (*χ*^2^ = 11.903, *p* = 0.001; *χ*^2^ = 36.830, *p* < 0.001; *χ*^2^ = 7.315, *p* = 0.007). Compared with conservative treatment, patients receiving TACE treatment after HCC rupture exhibited longer survival. The median survival time was 477 days for patients receiving TACE treatment after HCC rupture and 170 days for patients who underwent conservative treatment after HCC rupture (*p* < 0.001) (Fig. 2). Independent risk factors for overall survival in patients treated with surgery, TACE or conservative treatment at HCC rupture were also analyzed. The results are shown in the supplementary materials (Table S1, Table S2 and Table S3).

**Table 3:** Multivariate analysis of risk factors related to overall survival in patients with hepatocellular carcinoma rupture

Variables	Patients (*n* = 127)	HR	95% CI	*p*
Largest tumor size (cm)	8.39 ± 4.11	1.127	1.056–1.203	< 0.001
Tumor number	2.42 ± 1.55	1.126	0.915–1.385	0.263
BCLC				
A + B (control)	96	–	–	–
C	31	2.184	1.116–4.276	0.023
Treatment before rupture				
None	89	–	–	–
TACE	38	1.068	0.602–1.895	0.822
Treatment after rupture				
Conservative (control)	87	–	–	–
TACE	40	0.549	0.321–0.939	0.029
Treatment at rupture				
Conservative (control)	24	–	–	–
TACE/TAE	61	0.300	0.151–0.596	0.001
Surgery	42	0.196	0.091–0.425	< 0.001
ALB, g/L	31.94 ± 7.72	0.949	0.908–0.990	0.017
TBil, umol/L	27.53 ± 27.51	1.014	1.003–1.026	0.014
Cr, umol/L	82.22 ± 41.03	1.005	1.000–1.010	0.073
APTT, S	37.81 ± 6.89	0.947	0.894–1.004	0.067
INR,	1.33 ± 0.39	3.895	1.344–11.287	0.012
PTA, %	74.62 ± 21.14	1.012	0.991–1.033	0.263

*BCLC* Barcelona clinic liver cancer, *TACE* Transarterial chemoembolization, *TAE* Transcatheter arterial embolization, *ALB* Albumin, *TBil* Total bilirubin, *Cr* Creatinine, *APTT* Activated partial thromboplastin time, *INR* International normalized ratio, *PTA* Prothrombin activity

### Predictive power evaluation of the MELD, Child–Pugh and SPHR model scores for 30 day survival

**Fig. 3:**
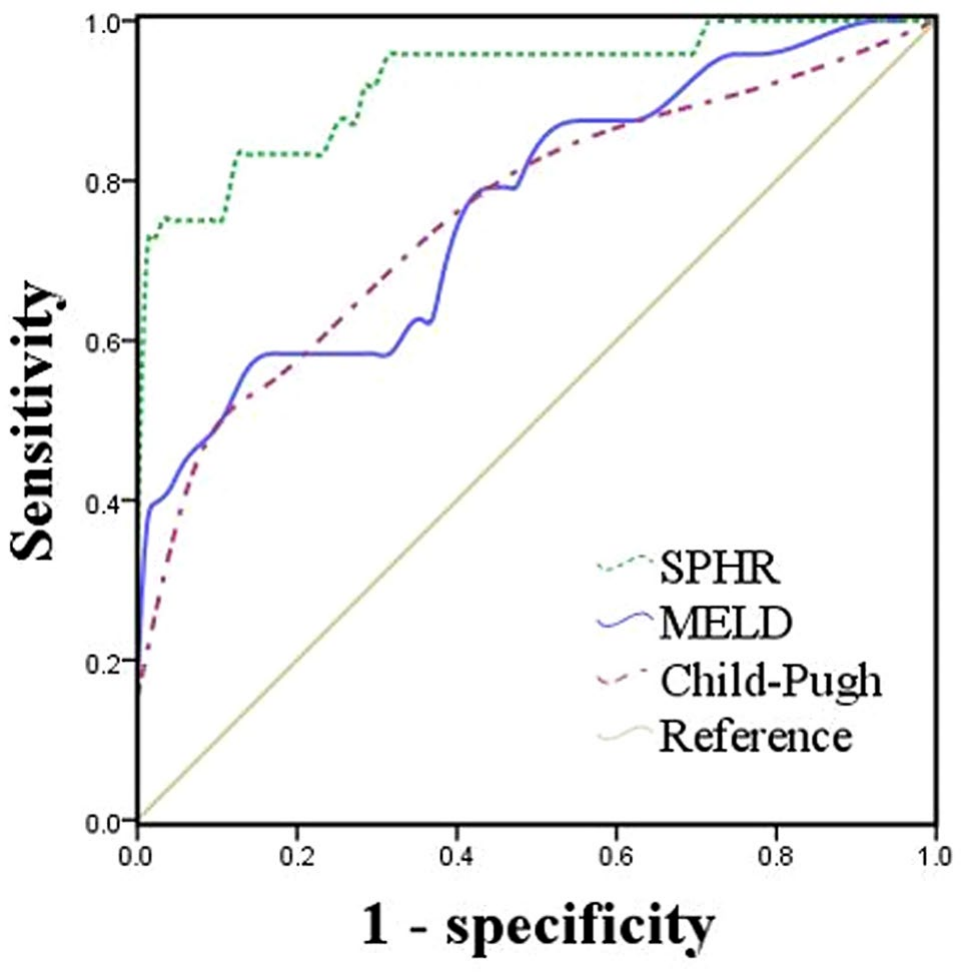
Receiver operating characteristic curve of the MELD score, Child–Pugh score and SPHR model. The area under the curve (AUC) of MELD was 0.767, the AUC of Child–Pugh was 0.757, and the AUC of SPHR was 0.925 (MELD vs. Child–Pugh: *p* = 0.849, MELD vs. SPHR: *p* = 0.010, Child–Pugh vs. SPHR: *p* = 0.002)

To investigate the predictive power of the MELD, Child–Pugh and SPHR model scores for 30 day survival, ROC curve analysis was conducted. The area under the curve (AUC) of the MELD score was 0.767, and the cutoff value for the MELD score was 13.4 for 30 day survival of HCC ruptured patients, with a sensitivity and specificity of 58.3% and 86.2%, respectively. In addition, the cutoff value for the MELD score was 9.0, with a sensitivity and specificity of 83.3% and 51.6%, respectively. The AUC of Child–Pugh score was 0.757, and the cutoff value was 8.5 for the 30 day survival of HCC ruptured patients, with a sensitivity and specificity of 54.2% and 84.2%, respectively. The SPHR model was described by the formula: *Y* = 0.078 × the largest tumor size + 0.250 × BCLC (A/B: 0, C: 1) + − 0.568 × TACE at rupture (No: 0, Yes: 1) + − 0.903 × Surgery at rupture (No: 0, Yes: 1) + 0.022 × ALB + 0.024 × TBil + 5.839 × INR–11.389. The AUC of SPHR was 0.925, the cutoff value for SPHR was 0.415, and the sensitivity and specificity were 75.0% and 97.9%, respectively. The AUC of the SPHR differed significantly from the AUC of the MELD score and Child–Pugh score (*p* = 0.010 and *p* = 0.002, respectively), and no significant difference in the AUC was found between the MELD score and Child–Pugh score (*p* = 0.849) (Fig. 3). Moreover, the predictability of SPHR in HBV and non-HBV HCC patients was evaluated, and the AUCs of SPHR in HBV and non-HBV HCC patients were 0.910 and 0.979, respectively (Supplementary Figure S1).

## Discussion

Spontaneous HCC rupture is a rare, life-threatening, and acute abdominal disease that accounts for 6–10% mortality in patients with HCC [8]. Various studies have demonstrated that HCC rupture may be attributed to increased intratumoral pressure, tumor size of > 5 cm, rapid growth of tumor volume, tumor necrosis, vessel obstruction by tumor thrombus, and subcapsular location [9–11]. However, factors related to patient survival still need further investigation. The present results showed that hepatocellular tumor size, treatment at rupture and in the follow-up, and hepatic function at rupture were significantly associated with survival following HCC rupture. In addition, the MELD score was relatively superior to the Child–Pugh score for predicting short-term survival without a significant difference. Furthermore, the SPHR model calculated in the present study showed a more accurate predictive efficacy for the short-term survival of HCC rupture.

The Child–Pugh score and MELD score are commonly used to assess liver function in patients with liver disease [12]. The Child–Pugh classification contains five variables, and two clinical determinants, ascites and encephalopathy, are based on subjective assessment [13]. The MELD score is based only on laboratory data, which should be more objective and accurate than the Child–Pugh score [14]. Previous studies have shown that the Child–Pugh score and MELD score are associated with the survival of patients with spontaneous HCC rupture [15–18], and our study demonstrated similar results. Furthermore, the predictive powers of both scores for 30 day survival were evaluated in our study, and the results showed that MELD was relatively superior to Child–Pugh for predicting short-term survival, although the difference was not significant. This result may be due to ascites evaluation in the Child–Pugh scoring system. From our perspective, intraperitoneal hematocele and infection caused by tumor rupture can stimulate the peritoneum to produce or increase ascites [19], which is different from the ascites caused by hepatic decompensation. Therefore, ascites as an index in HCC ruptured patients may not be accurate for evaluating hepatic function. Moreover, independent variables in multivariate analysis for overall survival were used to create a new predictive model, termed SPHR. The predictive value of the SPHR model was more accurate than the MELD score and Child–Pugh score for 30 day survival in patients. All of the abovementioned results may be helpful in patients’ clinical evaluation.

In the present study, TBil level (HR 1.014; *p* = 0.014) and INR level (HR 3.895; *p* = 0.012) were independent risk factors for overall survival of patients with HCC rupture in multivariate analysis. Moreover, TBil level and PT/INR level are variables contained in both the Child–Pugh and MELD systems and play an important role in influencing predictions of overall survival of patients [15, 20]. Therefore, the TBil level and PT/INR level of patients at HCC rupture merit greater emphasis in clinical practice. As confounding factors for TBil level and INR level, Child–Pugh and MELD were not included in the multivariate analysis. Our previous report showed that treatment before rupture was a risk factor related to overall survival [15], but this was not observed in the present study. This discrepancy may be due to the increased sample size and extended follow-up period. Cumulative survival analysis in the present study showed that patients with treatment before rupture demonstrated a significantly lower survival than patients without treatment before rupture within 500 days, which is similar to the previous study. However, with the extension of the follow-up period, the survival difference gradually lost its statistical significance (Supplementary Figure S2).

Acute and effective hemostasis is essential for the treatment of patients with HCC rupture. In the present study, surgery and TAE/TACE were proven to be more effective and beneficial therapies for HCC ruptured patients than conservative treatment. Similar to previous reports [21, 22], our results revealed that patients who received surgery achieved longer survival than patients who underwent TAE/TACE. TAE/TACE has been established as an effective, minimally invasive treatment for immediate hemostasis since the 1980s [4]. However, TAE/TACE for tumor treatment is less efficacious than surgery, and patients undergoing surgery often have better hepatorenal reservation. Additionally, the study by Chen et al. showed that spontaneous tumor rupture has no impact on perioperative morbidity or mortality after hepatectomy [23]. Thus, surgical and TACE/TAE treatment should be prioritized for patients with HCC rupture in the clinic. Moreover, it has been known that TACE is an effective strategy to control tumor growth in HCC patients, and it provides a better survival than supportive care treatment [24, 25]. The results of the present study showed similar findings, in that TACE was more effective than conservative treatment in the follow-up.

There are several limitations in this study. First, the study is inherently limited by its retrospective design. Second, the sample size of the present study was relatively small. Third, the validation of the SPHR model was not conducted in an independent cohort. Therefore, a large-scale, multicenter study may be warranted in the future. Moreover, the role of antiviral treatment on the prognosis of patients with HCC rupture was not investigated, and further effort will be needed in the future.

## Conclusion

Spontaneous rupture of HCC is a fatal condition with a poor prognosis. Our study demonstrated that the largest tumor size, BCLC stage, treatment at rupture, treatment after rupture, ALB level, TBil level, Cr level, and INR level were the most crucial predictors associated with overall survival. Additionally, the MELD score was relatively better for predicting 30 day survival in patients with HCC rupture than the Child–Pugh score without a significant difference, and the SPHR model was more valuable than the MELD score and Child–Pugh score for predicting 30 day survival in patients with HCC rupture.

## Supplementary Information

Below is the link to the electronic supplementary material.

Supplementary file1 (DOCX 222 KB)

## Data Availability

The datasets used and analyzed during the current study are available from the corresponding author on reasonable request.

## Abbreviations

AFP: Alpha-fetoprotein
ALB: Albumin
ALT: Alanine transaminase
APTT: Activated partial thromboplastin time
AUC: Area under the curve
BCLC: Barcelona clinic liver cancer
Cr: Creatinine
CT: Computed tomography
ECOG: Eastern cooperative oncology group
MRI: Magnetic resonance imaging
HB: Hemoglobin
HBV: Hepatitis B virus
HCC: Hepatocellular carcinoma
HCV: Hepatitis C virus
HR: Hazard ratio
INR: International normalized ratio
MELD: Model for end-stage liver disease
OS: Overall survival
PLT: Platelet count
PT: Prothrombin time
PTA: Prothrombin activity
RBC: Red blood cell count
ROC: Receiver operating characteristic
SPHR: Survival predictive model for HCC rupture
srHCC: Spontaneously ruptured hepatocellular carcinoma
TACE: Transarterial chemoembolization
TAE: Transcatheter arterial embolization
TBil: Total bilirubin
UICC: The Union for International Cancer Control
WBC: White blood cell count

## References

[1] Chen W, Zheng R, Baade PD (2016). Cancer statistics in China 2015. CA.

[2] Chung W, Jo C, Chung WJ (2018). Liver cirrhosis and cancer: comparison of mortality. Hepatol Int.

[3] Hsueh K-C, Fan H-L, Chen T-W (2012). Management of spontaneously ruptured hepatocellular carcinoma and hemoperitoneum manifested as acute abdomen in the emergency room. World J Surg.

[4] Yoshida H, Mamada Y, Taniai N (2016). Spontaneous ruptured hepatocellular carcinoma. Hepatol Res.

[5] Al-Mashat FM, Sibiany AM, Kashgari RH (2002). Spontaneous rupture of hepatocellular carcinoma. Saudi Med J.

[6] Angermayr B, Cejna M, Karnel F (2003). Child-Pugh versus MELD score in predicting survival in patients undergoing transjugular intrahepatic portosystemic shunt. Gut.

[7] Omata M, Cheng AL, Kokudo N (2017). Asia-Pacific clinical practice guidelines on the management of hepatocellular carcinoma: a 2017 update. Hepatol Int.

[8] Hong DF, Liu YB, Peng SY (2015). Management of hepatocellular carcinoma rupture in the caudate lobe. World J Gastroenterol.

[9] Tanaka T, Yamanaka N, Oriyama T (1997). Factors regulating tumor pressure in hepatocellular carcinoma and implications for tumor spread. Hepatology.

[10] Wu TH, Yu MC, Chen TC (2012). Encapsulation is a significant prognostic factor for better outcome in large hepatocellular carcinoma. J Surg Oncol.

[11] Tartaglia N, Di Lascia A, Cianci P (2020). Hemoperitoneum caused by spontaneous rupture of hepatocellular carcinoma in noncirrhotic liver. A case report and systematic review. Open Med (Warsaw, Poland).

[12] Wu JJ, Zhang ZG, Zhu P (2019). Comparative liver function models for ruptured hepatocellular carcinoma: a 10-year single center experience. Asian J Surg.

[13] Pugh RN, Murray-Lyon IM, Dawson JL (1973). Transection of the oesophagus for bleeding oesophageal varices. Br J Surg.

[14] Kamath PS, Wiesner RH, Malinchoc M (2001). A model to predict survival in patients with end-stage liver disease. Hepatology (Baltimore, MD).

[15] Han XJ, Su HY, Shao HB (2015). Prognostic factors of spontaneously ruptured hepatocellular carcinoma. World J Gastroenterol.

[16] Schwarz L, Bubenheim M, Zemour J (2018). Bleeding recurrence and mortality following interventional management of spontaneous HCC rupture: results of a multicenter European Study. World J Surg.

[17] Zhang XF, Wei T, Liu XM (2012). Spontaneous tumor rupture and surgical prognosis of patients with hepatocellular carcinoma. Scand J Gastroenterol.

[18] Jundt MC, Owen RL, Thompson SM (2022). MELD-Na > 16 is associated with high peri-procedural and short-term mortality in patients with ruptured hepatocellular carcinoma treated with emergent transarterial embolization. Abdom Radiol (NY).

[19] Mizuno S, Yamagiwa K, Ogawa T (2004). Are the results of surgical treatment of hepatocellular carcinoma poor if the tumor has spontaneously ruptured?. Scand J Gastroenterol.

[20] Sahu SK, Chawla YK, Dhiman RK (2019). Rupture of hepatocellular carcinoma: a review of literature. J Clin Exp Hepatol.

[21] Aoki T, Kokudo N, Matsuyama Y (2014). Prognostic impact of spontaneous tumor rupture in patients with hepatocellular carcinoma: an analysis of 1160 cases from a nationwide survey. Ann Surg.

[22] Zhang DZ, Zhang K, Wang XP (2015). Patients with spontaneously ruptured hepatocellular carcinoma benefit from staged surgical resection after successful transarterial embolization. APJCP.

[23] Chen Y, Guo D, Li X (2022). Predictors of spontaneous rupture of hepatocellular carcinoma and clinical outcomes following hepatectomy. Front Oncol.

[24] Han K, Kim JH (2015). Transarterial chemoembolization in hepatocellular carcinoma treatment: Barcelona clinic liver cancer staging system. World J Gastroenterol.

[25] de Baere T, Arai Y, Lencioni R (2016). Treatment of liver tumors with lipiodol TACE: technical recommendations from experts opinion. Cardiovasc Intervent Radiol.

