# Supplementary material for: Prognosis factors of predicting survival in spontaneously ruptured hepatocellular carcinoma
Source: Hepatol Int. 2022 Aug 25;16(6):1330–8. doi: 10.1007/s12072-022-10403-x (PMC9701175; doi:10.1007/s12072-022-10403-x)
Supplement: Supplementary file 1 — Supplementary file1 (DOCX 222 KB) [file 12072_2022_10403_MOESM1_ESM.docx]

**Prognosis factors of predicting survival in** **spontaneously ruptured** **h****epatocellular carcinoma**

Peng Wang^1^, Abraham S Moses^2^, Chao Li^1^, Song Chen^1^, Xun Qi^1^, Ke Xu^1^, Hai-bo Shao^1^, Xiang-jun Han^*1^

1 Department of Radiology, the First Affiliated Hospital of China Medical University, Shenyang, Liaoning, 110001, China

2 Department of Pharmaceutical Sciences, College of Pharmacy, Oregon State University, Portland, Oregon 97201, USA

**Table S1.** Univariate and multivariate analysis of risk factors related to overall survival in patients treated by surgery at HCC rupture

| **Variables** | **Univariate analysis** | | |  | **Multivariate analysis** | | |
| --- | --- | --- | --- | --- | --- | --- | --- |
|  | **HR** | **95% CI** | ***P*** |  | **HR** | **95% CI** | ***P*** |
| Age (years) | 0.997 | 0.955-1.042 | 0.904 |  |  |  |  |
| Gender | 0.980 | 0.420-2.286 | 0.962 |  |  |  |  |
| Largest tumor size (cm) | 1.105 | 1.016-1.201 | **0.019** |  | 1.153 | 1.052-1.264 | **0.002** |
| Tumor number | 1.224 | 0.894-1.676 | 0.207 |  |  |  |  |
| Tumor size | 1.357 | 0.597-3.084 | 0.466 |  |  |  |  |
| Up-to-seven* | 2.871 | 1.178-6.997 | **0.020** |  |  |  |  |
| Tumor rupture location  Left lobe (control)  Right lobe | -  0.549 | -  0.241-1.248 | -  0.152 |  |  |  |  |
| BCLC  A+B (control)  C | -  1.579 | -  0.458-5.445 | **-**  0.469 |  |  |  |  |
| UICC TNM stage  ⅢC (control)  ⅣA+ⅣB | -  2.364 | -  0.928-6.024 | -  0.071 |  |  |  |  |
| Treatment before rupture | 0.318 | 0.074-1.363 | 0.123 |  |  |  |  |
| Treatment after rupture  Conservative (control)  TACE | -  0.879 | -  0.391-1.975 | **-**  0.755 |  |  |  |  |
| Virus  None (control)  HBV | -  0.566 | -  0.191-1.679 | 0.305 |  |  |  |  |
| Heart rate | 0.993 | 0.969-1.018 | 0.578 |  |  |  |  |
| Transfusion | 1.145 | 0.498-2.633 | 0.750 |  |  |  |  |
| AFP, ng/mL  ≤400 (control)  >400 | -  1.568 | -  0.702-3.503 | -  0.273 |  |  |  |  |
| WBC, ×10^9^/L | 1.015 | 0.952-1.082 | 0.646 |  |  |  |  |
| RBC, ×10^12^/L | 0.844 | 0.499-1.426 | 0.526 |  |  |  |  |
| HB, g/L | 0.993 | 0.976-1.011 | 0.456 |  |  |  |  |
| PLT, ×10^9^/L | 1.001 | 0.995-1.007 | 0.682 |  |  |  |  |
| PT, S | 1.120 | 0.931-1.347 | 0.231 |  |  |  |  |
| INR | 9.845 | 2.135-45.391 | **0.003** |  | 18.276 | 3.423-97.588 | **0.001** |
| APTT, S | 1.033 | 0.949-1.123 | 0.456 |  |  |  |  |
| PTA, % | 0.997 | 0.975-1.019 | 0.794 |  |  |  |  |
| ALT, U/L | 1.001 | 0.997-1.005 | 0.652 |  |  |  |  |
| ALB, g/L | 0.958 | 0.904-1.016 | 0.155 |  |  |  |  |
| TBil, umol/L | 1.011 | 0.995-1.027 | 0.163 |  |  |  |  |
| K^+^, mmol/L | 1.415 | 0.685-2.923 | 0.348 |  |  |  |  |
| Cr, umol/L | 1.009 | 0.998-1.020 | 0.119 |  |  |  |  |
| Child-Pugh score | 1.226 | 0.950-1.581 | 0.118 |  |  |  |  |
| MELD score | 1.104 | 0.986-1.236 | 0.087 |  |  |  |  |

Note: BCLC: Barcelona Clinic Liver Cancer; UICC: The Union for International Cancer Control; TACE: Transarterial chemoembolization; HBV: Hepatitis B virus; AFP: alpha-fetoprotein; WBC: White blood cell count; RBC: Red blood cell count; HB: hemoglobin; PLT: Platelet count; PT: prothrombin time; INR: International normalized ratio; APTT: Activated partial thromboplastin time; PTA: Prothrombin activity; ALT: Alanine transaminase; ALB: Albumin; TBil: Total bilirubin; Cr: Creatinine. MELD: Model for end-stage liver disease.

*Up-to-seven is a confounding factor for the largest tumor size and not included in multivariable analysis.

**Table S2.** Univariate and multivariate analysis of risk factors related to overall survival in patients treated by TACE/TAE at HCC rupture

| **Variables** | **Univariate analysis** | | |  | **Multivariate analysis** | | |
| --- | --- | --- | --- | --- | --- | --- | --- |
|  | **HR** | **95% CI** | ***P*** |  | **HR** | **95% CI** | ***P*** |
| Age (years) | 0.999 | 0.970-1.029 | 0.949 |  |  |  |  |
| Gender | 1.032 | 0.245-4.340 | 0.966 |  |  |  |  |
| Largest tumor size (cm) | 1.107 | 1.017-1.206 | **0.019** |  | 1.028 | 0.917-1.151 | 0.639 |
| Tumor number | 1.408 | 1.109-1.788 | **0.005** |  | 1.289 | 0.960-1.729 | 0.091 |
| Tumor size | 1.417 | 0.621-3.230 | 0.408 |  |  |  |  |
| Up-to-seven | 2.294 | 0.997-5.276 | 0.051 |  |  |  |  |
| Tumor rupture location  Left lobe (control)  Right lobe | -  1.076 | -  0.517-2.239 | -  0.845 |  |  |  |  |
| BCLC  A+B (control)  C | -  4.389 | -  1.961-9.822 | **-**  **<0.001** |  | 3.825 | 1.546-9.563 | **0.004** |
| UICC TNM stage  ⅢC (control)  ⅣA+ⅣB | -  1.320 | 0.649-2.687 | 0.443 |  |  |  |  |
| Treatment before rupture | 1.342 | 0.683-2.638 | 0.393 |  |  |  |  |
| Treatment after rupture  Conservative (control)  TACE | -  0.416 | -  0.200-0.864 | **-**  **0.019** |  | 0.460 | 0.202-1.047 | 0.064 |
| Virus  None (control)  HBV  HCV | -  1.533  1.219 | -  0.464-5.068  0.124-11.951 | -  0.484  0.865 |  |  |  |  |
| Heart rate | 1.002 | 0.986-1.018 | 0.811 |  |  |  |  |
| Transfusion | 0.630 | 0.241-1.645 | 0.345 |  |  |  |  |
| AFP, ng/mL  ≤400 (control)  >400 | -  1.446 | -  0.752-2.781 | -  0.269 |  |  |  |  |
| WBC, ×10^9^/L | 1.003 | 0.954-1.055 | 0.892 |  |  |  |  |
| RBC, ×10^12^/L | 1.272 | 0.882-1.834 | 0.197 |  |  |  |  |
| HB, g/L | 1.005 | 0.992-1.019 | 0.445 |  |  |  |  |
| PLT, ×10^9^/L | 1.002 | 0.997-1.007 | 0.452 |  |  |  |  |
| PT, S | 0.978 | 0.844-1.134 | 0.770 |  |  |  |  |
| INR | 4.684 | 1.627-13.484 | **0.004** |  | 4.005 | 1.076-14.902 | **0.038** |
| APTT, S | 1.032 | 0.979-1.089 | 0.242 |  |  |  |  |
| PTA, % | 0.993 | 0.977-1.011 | 0.451 |  |  |  |  |
| ALT, U/L | 1.003 | 1.001-1.006 | **0.014** |  | 1.003 | 1.000-1.006 | 0.097 |
| ALB, g/L | 0.966 | 0.926-1.007 | 0.106 |  |  |  |  |
| TBil, umol/L | 1.027 | 1.003-1.051 | **0.024** |  | 1.007 | 0.980-1.035 | 0.606 |
| K^+^, mmol/L | 0.762 | 0.403-1.444 | 0.405 |  |  |  |  |
| Cr, umol/L | 1.002 | 0.996-1.009 | 0.435 |  |  |  |  |
| Child-Pugh score | 1.097 | 0.907-1.326 | 0.340 |  |  |  |  |
| MELD score | 1.037 | 0.957-1.124 | 0.371 |  |  |  |  |

Note: BCLC: Barcelona Clinic Liver Cancer; UICC: The Union for International Cancer Control; TACE: Transarterial chemoembolization; HBV: Hepatitis B virus; HCV: Hepatitis C virus; AFP: alpha-fetoprotein; WBC: White blood cell count; RBC: Red blood cell count; HB: hemoglobin; PLT: Platelet count; PT: prothrombin time; INR: International normalized ratio; APTT: Activated partial thromboplastin time; PTA: Prothrombin activity; ALT: Alanine transaminase; ALB: Albumin; TBil: Total bilirubin; Cr: Creatinine. MELD: Model for end-stage liver disease.

**Table S3.** Univariate and multivariate analysis of risk factors related to overall survival in patients treated by conservative at HCC rupture

| **Variables** | **Univariate analysis** | | |  | **Multivariate analysis** | | |
| --- | --- | --- | --- | --- | --- | --- | --- |
|  | **HR** | **95% CI** | ***P*** |  | **HR** | **95% CI** | ***P*** |
| Age (years) | 0.980 | 0.941-1.021 | 0.341 |  |  |  |  |
| Gender | 0.991 | 0.378-2.602 | 0.986 |  |  |  |  |
| Largest tumor size (cm) | 1.147 | 1.004-1.310 | **0.043** |  | 1.181 | 0.975-1.430 | 0.089 |
| Tumor number | 1.515 | 1.087-2.112 | **0.014** |  | 1.414 | 0.904-2.414 | 0.129 |
| Tumor size | 2.134 | 0.693-6.574 | 0.186 |  |  |  |  |
| Up-to-seven | 52.632 | - | 0.067 |  |  |  |  |
| Tumor location  Left lobe (control)  Right lobe | -  1.521 | -  0.617-3.751 | -  0.363 |  |  |  |  |
| BCLC  A+B (control)  C | -  1.962 | -  0.729-5.280 | **-**  0.182 |  |  |  |  |
| UICC TNM stage  ⅢC (control)  ⅣA+ⅣB | -  4.878 | -  1.462-16.278 | **-**  **0.010** |  | 3.519 | 0.733-16.907 | 0.116 |
| Treatment before rupture | 2.048 | 0.808-5.194 | 0.131 |  |  |  |  |
| Treatment after rupture  Conservative (control)  TACE | -  0.080 | -  0.010-0.629 | **-**  **0.016** |  | 0.082 | 0.006-1.203 | 0.068 |
| Virus  None (control)  HBV  HCV | -  1.607  4.667 | -  0.456-5.666  0.675-32.246 | -  0.461  0.118 |  |  |  |  |
| Heart rate | 1.019 | 0.989-1.050 | 0.226 |  |  |  |  |
| Transfusion | 1.206 | 0.389-3.744 | 0.746 |  |  |  |  |
| AFP, ng/mL  ≤400 (control)  >400 | -  2.033 | -  0.708-5.836 | -  0.187 |  |  |  |  |
| WBC, ×10^9^/L | 1.068 | 0.932-1.224 | 0.344 |  |  |  |  |
| RBC, ×10^12^/L | 0.732 | 0.474-1.131 | 0.160 |  |  |  |  |
| HB, g/L | 0.992 | 0.977-1.007 | 0.303 |  |  |  |  |
| PLT, ×10^9^/L | 1.000 | 0.996-1.005 | 0.912 |  |  |  |  |
| PT, S* | 1.149 | 1.040-1.270 | **0.007** |  |  |  |  |
| INR | 4.058 | 1.636-10.067 | **0.003** |  | 0.564 | 0.128-2.482 | 0.449 |
| APTT, S | 1.048 | 0.994-1.105 | 0.081 |  |  |  |  |
| PTA, % | 0.984 | 0.969-1.000 | 0.050 |  |  |  |  |
| ALT, U/L | 0.998 | 1.000-1.002 | 0.102 |  |  |  |  |
| ALB, g/L | 0.943 | 0.885-1.005 | 0.072 |  |  |  |  |
| TBil, umol/L | 1.028 | 1.013-1.044 | **<0.001** |  | 1.035 | 1.012-1.059 | **0.002** |
| K^+^, mmol/L | 0.861 | 0.372-1.992 | 0.726 |  |  |  |  |
| Cr, umol/L | 1.004 | 0.992-1.016 | 0.546 |  |  |  |  |
| Child-Pugh score* | 1.517 | 1.173-1.962 | **0.001** |  |  |  |  |
| MELD score* | 1.129 | 1.044-1.221 | **0.002** |  |  |  |  |

Note: BCLC: Barcelona Clinic Liver Cancer; UICC: The Union for International Cancer Control; TACE: Transarterial chemoembolization; HBV: Hepatitis B virus; HCV: Hepatitis C virus; AFP: alpha-fetoprotein; WBC: White blood cell count; RBC: Red blood cell count; HB: hemoglobin; PLT: Platelet count; PT: prothrombin time; INR: International normalized ratio; APTT: Activated partial thromboplastin time; PTA: Prothrombin activity; ALT: Alanine transaminase; ALB: Albumin; TBil: Total bilirubin; Cr: Creatinine. MELD: Model for end-stage liver disease.

*PT is a confounding factor for INR and not included in multivariable analysis; Child-Pugh score and MELD score, as confounding factors for INR and TBil, are not included in multivariable analysis.


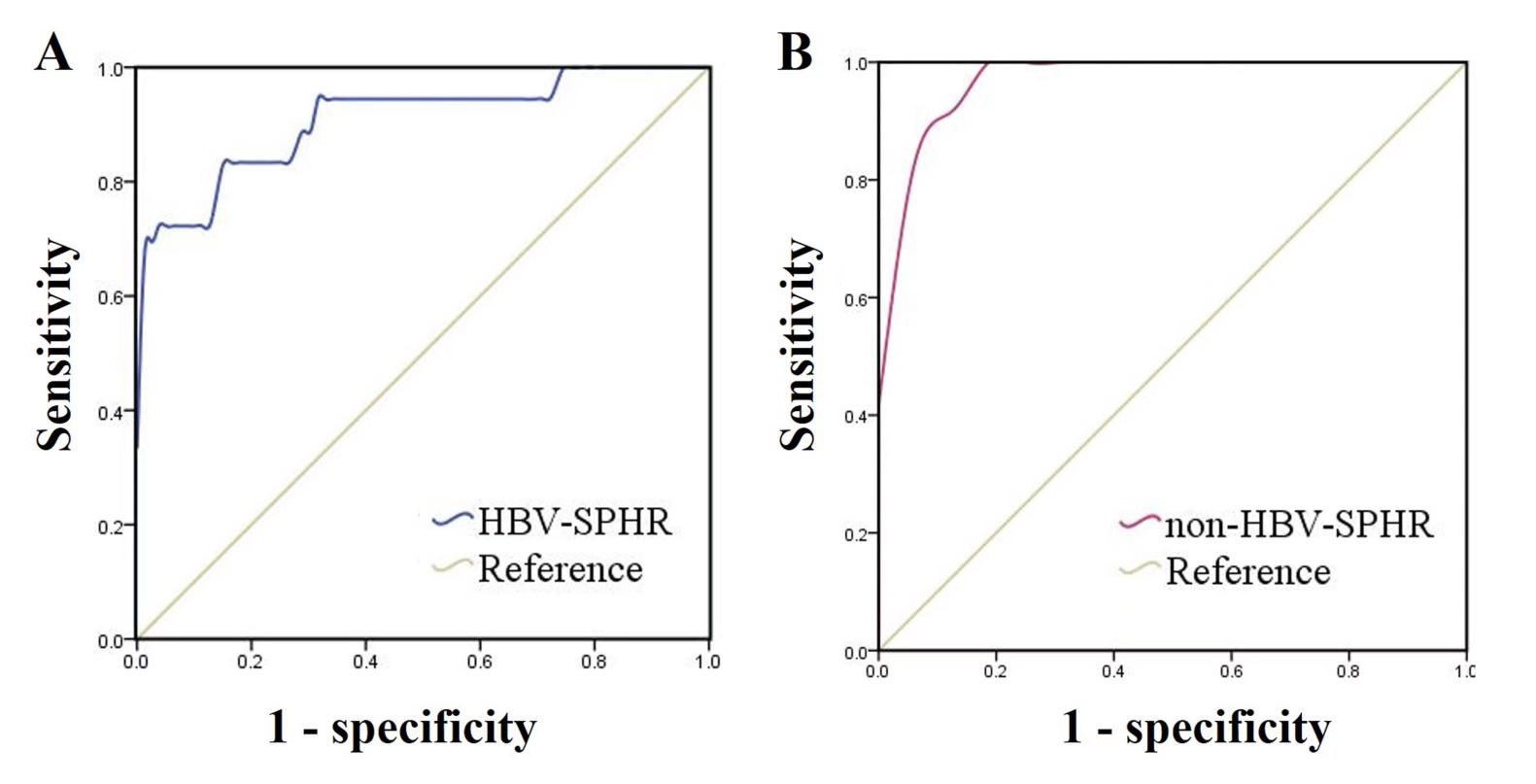


**Figure S1. Receiver operating characteristic (ROC) curve of SPHR model in HBV and non-HBV HCC patients.** A: ROC curve of SPHR model in HBV HCC patients, the area under the curve (AUC) was 0.910. B: ROC curve of SPHR model in non-HBV HCC patients, the AUC was 0.979.


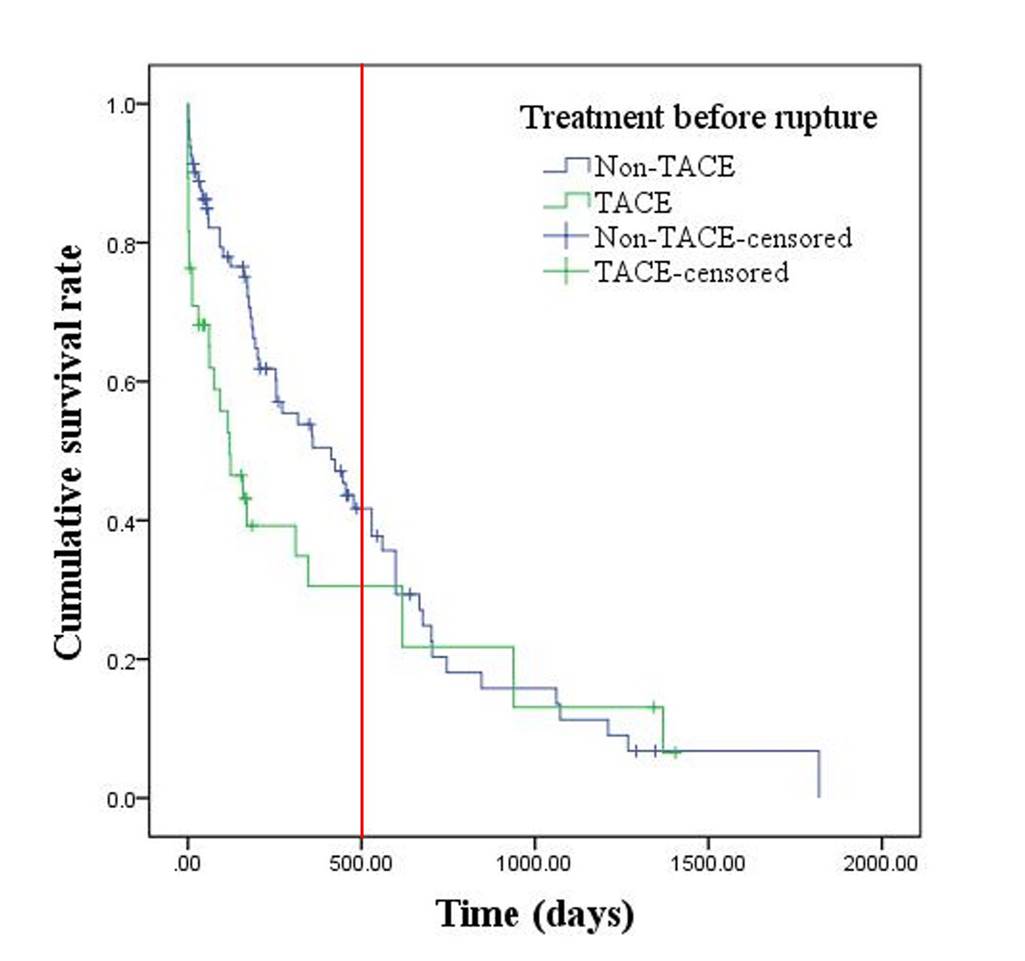


**Figure S2: Cumulative survival rate according to treatment before HCC rupture.** Patients with TACE before HCC rupture demonstrated a significantly lower cumulative survival rate than non-TACE patients within 500 days. With the follow-up of over than 500 days, the survival difference gradually lost its statistical significance (*P* = 0.133). Of 38 patients who received TACE/TAE before HCC rupture, 15 (39.5%) patients received repeated TACE at HCC rupture, 1 (2.6%) patients received TACE in the follow-up, and 5 (13.2%) patients received TACE both at tumor rupture and in the follow-up, others received conservative treatment.
